# Various *Bacillus* and *Paenibacillus* Spp. Isolated From Soil Produce Compounds With Potent Antimicrobial Activity Against Clinically Relevant Pathogens

**DOI:** 10.1002/mbo3.70179

**Published:** 2025-12-11

**Authors:** Michael Moran, Hogan Turner, Joseph Yanchar, Joshua Preece, Gene Ahlborn, Richard Robison

**Affiliations:** ^1^ Department of Microbiology and Molecular Biology Brigham Young University Provo Utah USA; ^2^ Department of Nutrition, Dietetics, and Food Science Brigham Young University Provo Utah USA

**Keywords:** antimicrobial compounds, antimicrobial resistance, carbapenem‐resistant Enterobacterales, genome mining, *Paenibacillus profundus*, soil bacteria

## Abstract

The increasing prevalence of antibiotic resistance among clinically significant pathogens necessitates the discovery of novel antimicrobial agents. This study investigated 29 *Bacillus* and *Paenibacillus* isolates from the soil for antimicrobial activity against multidrug‐resistant clinical pathogens, including methicillin‐resistant *Staphylococcus aureus* (MRSA) and carbapenem‐resistant Enterobacterales (CRE). In both agar‐ and broth‐based antimicrobial assays, *Paenibacillus profundus* strains 7.5 and M4.5 exhibited potent broad‐spectrum activity, including significant inhibition of many CREs. Species identification was performed through 16S rRNA sequencing, and genome mining of three producer strains using antiSMASH revealed biosynthetic gene clusters associated with a variety of nonribosomal peptide synthetases (NRPSs), polyketide synthases (PKSs), and ribosomally synthesized and post‐translationally modified peptides (RiPPs). While many of these clusters were not associated with known antimicrobial compounds, several of them displayed high similarity to known compounds such as polymyxin B, paenilan, colistin, and paenibacterin. These findings reinforce numerous previous studies highlighting the potential of soil‐derived *Bacillus* and *Paenibacillus* species as valuable sources of novel antimicrobials to address the global antibiotic resistance crisis.

## Graphical abstract

This study identified soil‐derived *Bacillus* and *Paenibacillus* strains with broad‐spectrum antimicrobial activity, including 97% inhibition of CREs by *Paenibacillus profundus*. Genome mining revealed biosynthetic gene clusters (NRPSs, PKSs, RiPPs) linked to antimicrobial production, highlighting their potential in combating antibiotic resistance. Created in BioRender. Moran, M. (2025) https://BioRender.com/o79z100

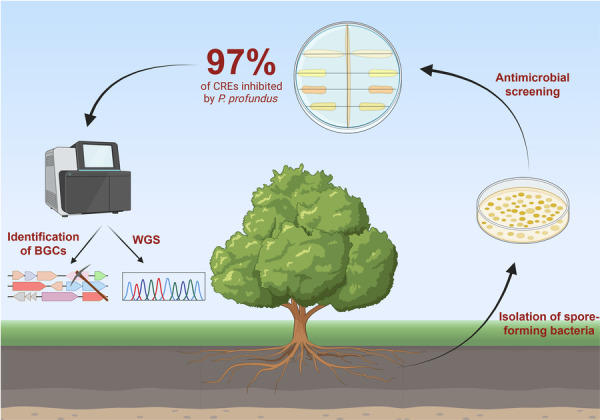

## 1 Introduction

Antibiotic resistances have been a problem since the inception of antibiotics nearly a century ago. Treatment of antibiotic‐resistant infections cost the U.S. an estimated $4.6 billion in 2017 (Nelson et al. 2021). Additionally, it is predicted that drug‐resistant infections will cause 10 million deaths per year worldwide by 2050 (De Kraker et al. 2016). Despite these dire circumstances, the FDA has only approved a handful of new antibiotics in recent years, largely due to the high costs and complex regulatory hurdles associated with drug development (Dutescu and Hillier 2021). Consequently, there is an urgent need to discover or synthesize novel antimicrobials effective against drug‐resistant pathogens. To achieve this, it is essential to account for the intrinsic, genetic, and nongenetic mechanisms that underlie bacterial resistance.

**Figure 1:**
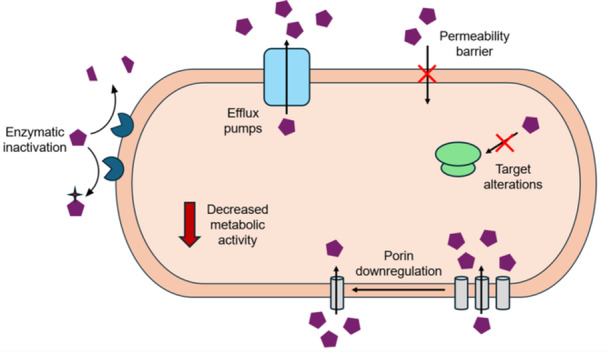
Mechanisms of antibiotic resistance in bacteria. Bacteria have evolved many different strategies to protect themselves from the effects of antibiotics. Permeability barriers are intrinsic to the structure of the bacteria and provide resistance to specific groups of antibiotics. Porins in the membrane can be downregulated to decrease drug permeability. Some bacteria carry genes that encode enzymes that inactivate antibiotics or alter their targets, as well as increase drug efflux. Decreased metabolic activity is an example of a nongenetic mechanism of resistance.

Bacteria employ a variety of mechanisms to resist the effects of antibiotics, one of which is intrinsic resistance (Figure 1). A prominent example of intrinsic resistance is the presence of a hydrophobic outer membrane (OM) in Gram‐negative bacteria. This membrane serves as a permeability barrier that restricts many antibiotic classes from reaching their intracellular targets (Breijyeh et al. 2020), including bulky molecules such as vancomycin (Exner et al. 2017). The permeability of the OM varies among Gram‐negative bacteria. The hydrophobic portion of the OM, known as lipid A, frequently undergoes structural modifications that can alter membrane permeability (Needham and Trent 2013; Matsuura 2013; Gupta and Datta 2019). While the OM permits the diffusion of hydrophobic antibiotics like macrolides, small hydrophilic antibiotics such as β‐lactams must traverse outer membrane proteins (OMPs) to access their targets (James et al. 2009; Delcour 2009). Porins, a type of OMP that facilitate the influx of hydrophilic solutes, vary widely among Gram‐negative species in their structure, selectivity, and abundance (Ghai 2023; Pagès et al. 2008), further influencing antibiotic susceptibility. Moreover, bacteria can modify their porin expression or specificity in response to antibiotic stress (Pagès et al. 2008). Intrinsic resistance is also seen in Gram‐positive bacteria. For example, these bacteria are largely unaffected by polymyxins because they lack lipid A, which is the primary target for this class of antibiotics (Mohapatra et al. 2021; Yin et al. 2020).

In addition to intrinsic factors, genetic factors also play a major role in antimicrobial resistance among bacteria. Horizontal gene transfer enables bacteria to acquire mobile genetic elements (MGEs) from both the environment and other microbial species (Tokuda and Shintani 2024). MGEs can contribute to antibiotic resistance by carrying genes that encode enzymes which modify or degrade antibiotics, alter their cellular targets, or increase drug efflux (Breijyeh et al. 2020; Miller 2016; Gauba and Rahman 2023). These elements can be exchanged not only among members of the same species but also across different species and genera (Exner et al. 2017).

Bacteria can also employ nongenetic mechanisms that confer transient, non‐heritable resistance to antibiotics (Corona and Martinez 2013). One such mechanism is biofilm formation, which reduces susceptibility by limiting antibiotic penetration and by slowing or arresting bacterial growth within the biofilm (Belay et al. 2024; Sharma et al. 2023). Bacteria that remain genetically susceptible yet exhibit altered metabolic activity that protects them from antibiotic effects are known as persister cells, and they can arise both within and outside of biofilms (Corona and Martinez 2013; Niu et al. 2024).

The complexity and diversity of bacterial resistance mechanisms highlight the challenges in developing effective new antibiotics. In response, researchers have historically looked to soil microbes, which have spent millions of years competing with one another, as a rich source of potential antibiotic compounds. Despite decades of exploration, the immense biodiversity within soil ecosystems continues to present opportunities for discovering novel antimicrobial molecules capable of overcoming existing resistance barriers.

Two groups of soil bacteria that are known for their prolific production of antimicrobial compounds are *Bacillus* and *Paenibacillus* (Song 2025; Harwood et al. 2018; Grady et al. 2016; Olishevska et al. 2019; Celandroni et al. 2016). While some of their produced compounds are currently in use, such as bacitracin from *Bacillus subtilis* (Johnson et al. 1945) and polymyxin B and E from *Paenibacillus polymyxa* (Al‐Thubiani et al. 2018; Ainsworth et al. 1947), there may be uncharacterized antimicrobial compounds encoded by these bacteria that have potential for clinical, industrial, and agricultural use. These compounds are synthesized through a variety of means within the bacterium, including via ribosomes and complex enzymes such as nonribosomal peptide synthetases (NRPS) and polyketide synthases (PKS) (Scherlach and Hertweck 2021). Genome mining involves the *in silico* analysis of microbial genomes in search of biosynthetic gene clusters (such as NRPS) that encode genes involved in the production of antimicrobial compounds, followed by in vitro analysis of the gene products (Mokhtar et al. 2020). Genome mining can lead to discovery of both known and unknown antimicrobial compounds (Scherlach and Hertweck 2021; Waongo et al. 2025; Yang et al. 2021; Lebedeva et al. 2021; Kim et al. 2024; Chunduru et al. 2024).

In addition to their well‐known capacity to produce antibacterial compounds, members of the *Bacillus* and *Paenibacillus* genera are spore‐forming bacteria, allowing their selective isolation from soil through heat treatment of samples containing complex microbial communities. This study investigated the antimicrobial activity of 29 *Bacillus* and *Paenibacillus* strains isolated from soil against a diverse panel of clinically relevant pathogens, many of which are considered priority pathogens by the WHO (WHO 2024). The activity of each isolate was measured using a cross‐streak method, and species‐level identification was carried out through 16S rRNA sequencing. The genomes of several isolates were also analyzed using the bioinformatics tool antiSMASH to identify biosynthetic gene clusters. Finally, cell‐free supernatant was extracted from the producer strains and tested for its antimicrobial activity. The results of this work demonstrate that several of the identified producer strains synthesize antimicrobial compounds that are capable of inhibiting the growth of clinically relevant, antibiotic‐resistant pathogens.

## 2 Materials and Methods

### 2.1 Strains and Growth Conditions

Producer strains (*Bacillus* and *Paenibacillus* spp. investigated for antimicrobial production) were grown on lysogeny broth (LB) agar plates at 30°C. Target strains (except for *Mycobacterium phlei*) were grown on LB agar plates at 37°C, and overnight cultures of target strains were grown in LB broth at 37°C while shaking at 175 rpm (Table 1). *M. phlei* was grown on LB agar at 30°C and overnight cultures were grown in nutrient broth supplemented with 2% Tween‐80 at 37°C while shaking at 175 rpm. Carbapenem‐resistant Enterobacterales (CRE) and methicillin‐resistant *Staphylococcus aureus* (MRSA) target strains were handled under enhanced biosafety level 2 (BSL‐2 +) conditions, and all other bacterial cultures were handled under BSL‐2 conditions. Freezer stocks of all strains were made with LB + 20% glycerol and stored at −20°C. LB and nutrient broth were purchased from Fisher Scientific and made according to manufacturer instructions. All media was sterilized by autoclaving before use.

**Table 1:** Target strains and growth conditions.

AR Bank^a^ # (if applicable)	Target strain	Source	Media/growth conditions
—	*Staphylococcus aureus* ATCC 6538	ATCC^b^	LB/37°C
—	*Escherichia coli* ATCC 11229	ATCC	LB/37°C
—	*Mycobacterium phlei* Presque Isle	Presque Isle Company	NB/37°C
—	*Pseudomonas aeruginosa* ATCC 15442	ATCC	LB/37°C
—	Methicillin‐resistant *S. aureus* (MRSA) ATCC 43300^g^	ATCC	LB/37°C
—	MRSA‐1	BYUA^c^	LB/37°C
—	MRSA‐2	BYUA	LB/37°C
—	MRSA‐3	BYUA	LB/37°C
—	MRSA‐4	BYUA	LB/37°C
—	MRSA‐5	BYUA	LB/37°C
—	MRSA‐6	BYUA	LB/37°C
—	MRSA‐7	BYUA	LB/37°C
—	MRSA‐8	BYUA	LB/37°C
—	MRSA‐9	BYUA	LB/37°C
1	*E. coli*	BIT^d^	LB/37°C
2	*Enterobacter cloacae*	BIT	LB/37°C
3	*Klebsiella pneumoniae*	BIT	LB/37°C
4	*K. pneumoniae*	BIT	LB/37°C
5	*K. pneumoniae*	BIT	LB/37°C
6	*E. coli*	BIT	LB/37°C
7	*Klebsiella aerogenes*	BIT	LB/37°C
8	*E. cloacae*	BIT	LB/37°C
9	*K. aerogenes*	BIT	LB/37°C
10	*K. pneumoniae*	BIT	LB/37°C
11	*E. coli*	BIT	LB/37°C
12	*K. pneumoniae*	BIT	LB/37°C
13	*E. coli*	BIT	LB/37°C
14	*E. coli*	BIT	LB/37°C
15	*E. coli*	BIT	LB/37°C
16	*K. pneumoniae*	BIT	LB/37°C
17	*E. coli*	BIT	LB/37°C
18	*K. aerogenes*	BIT	LB/37°C
19	*E. coli*	BIT	LB/37°C
20	*E. coli*	BIT	LB/37°C
21	*Citrobacter freundii*	BIT	LB/37°C
22	*C. freundii*	BIT	LB/37°C
23	*C. freundii*	BIT	LB/37°C
24	*Citrobacter koseri*	BIT	LB/37°C
25	*C. koseri*	BIT	LB/37°C
26	*Providencia stuartii*	BIT	LB/37°C
27	*Serratia marcescens*	BIT	LB/37°C
28	*Klebsiella oxytoca*	BIT	LB/37°C
29	*Proteus mirabilis*	BIT	LB/37°C
30	*Shigella sonnei*	BIT	LB/37°C
31	*Salmonella* Typhimurium	BIT	LB/37°C
40	*K. pneumoniae*	CarbaNP^e^	LB/37°C
46	*K. pneumoniae*	CarbaNP	LB/37°C
47	*K. pneumoniae*	CarbaNP	LB/37°C
56	*Acinetobacter baumannii*	CarbaNP	LB/37°C
57	*Morganella morganii*	CarbaNP	LB/37°C
59	*P. mirabilis*	CarbaNP	LB/37°C
62	*K. aerogenes*	CarbaNP	LB/37°C
73	*E. cloacae*	CarbaNP	LB/37°C
82	*Providencia rettgeri*	CarbaNP	LB/37°C
87	*K. pneumoniae*	CarbaNP	LB/37°C
91	*S. marcescens*	CarbaNP	LB/37°C
97	*K. pneumoniae*	CarbaNP	LB/37°C
99	*S. marcescens*	CarbaNP	LB/37°C
106	*K. pneumoniae*	CarbaNP	LB/37°C
109	*K. pneumoniae*	CarbaNP	LB/37°C
121	*S. marcescens*	CRE^f^	LB/37°C
122	*S. marcescens*	CRE	LB/37°C
123	*S. marcescens*	CRE	LB/37°C
124	*S. marcescens*	CRE	LB/37°C
125	*K. pneumoniae*	CRE	LB/37°C
130	*S. marcescens*	CRE	LB/37°C
131	*S. marcescens*	CRE	LB/37°C
133	*M. morganii*	CRE	LB/37°C
134	*Raoultella ornithinolytica*	CRE	LB/37°C
155	*P. mirabilis*	CRE	LB/37°C
156	*P. mirabilis*	CRE	LB/37°C
159	*P. mirabilis*	CRE	LB/37°C
163	*E. cloacae*	CRE	LB/37°C
507	*K. pneumoniae*	IMR^g^	LB/37°C
517	*S. marcescens*	IMR	LB/37°C
519	*M. morganii*	IMR	LB/37°C
520	*S. marcescens*	IMR	LB/37°C
521	*S. marcescens*	IMR	LB/37°C
522	*K. pneumoniae*	IMR	LB/37°C
523	*K. pneumoniae*	IMR	LB/37°C
524	*K. pneumoniae*	IMR	LB/37°C
525	*K. pneumoniae*	IMR	LB/37°C

^a^ CDC & FDA Antibiotic Resistant Isolate Bank.

^b^ American Type Culture Collection.

^c^ BYU Archive.

^d^ CDC Antibiotic Resistance Enterobacterales Carbapenem Breakpoint Panel.

^e^ CDC Antibiotic Resistance Gram Negative Carbapenemase Detection Panel.

^f^ CDC Antibiotic Resistance Enterobacterales Carbapenemase Diversity Panel.

^g^ CDC Antibiotic Resistance Imipenem/relebactam Panel.

### 2.2 Isolation of Spore‐Forming Bacteria From the Soil

Spore‐forming bacteria were isolated from the soil around the Brigham Young University campus in Provo, Utah County, Utah, USA. One g of each soil sample was suspended in 9 mL of purified water and then heated at 80°C for 10 min in a water bath to select for spore‐forming bacteria. Upon removal from the water bath, the soil suspensions were serially diluted in deionized water and 0.1 mL was plated on LB agar. A control plate was also prepared using 0.1 mL purified water. The plates were incubated for three to 7 days at 30°C. Colonies with unique morphologies were picked and streaked onto fresh LB agar plates and then incubated for approximately 48 h at 30°C.

### 2.3 Screening of Spore‐Forming Isolates for Antimicrobial Potential

**Figure 2:**
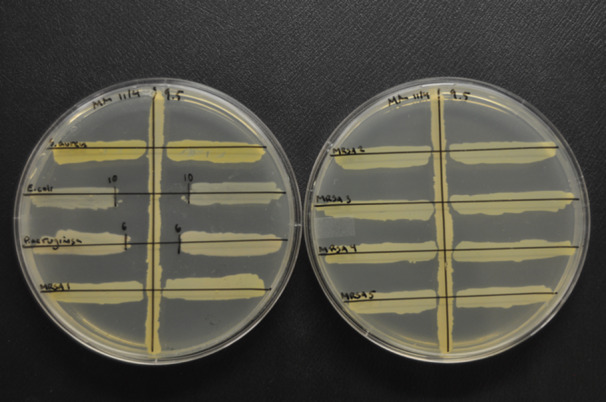
Example of the cross‐streak method. A single colony of the producer strain (*P. amylolyticus* 9.5) is inoculated as a single vertical line on the plate. After incubating the growth line for 48 h, overnight cultures of the target strains (*S. aureus*, *E. coli*, *P. aeruginosa*, MRSA #1‐5) are streaked perpendicular to the producer strain. The antimicrobial activity of the producer strain against the target strain is measured in mm after 24 and 48 h of incubation.

About 500 spore‐forming isolates isolated from the soil were initially screened for antimicrobial activity using an adapted spread‐patch method (Marcolefas et al. 2019) in which a colony from each strain was spread in a small patch (5–8 mm) on an LB agar plate containing a freshly plated liquid culture of a target strain. This method was used to collect preliminary data about promising antimicrobial producers, but it was replaced by a modified cross‐streak method (Carvajal 1947) primarily because the cross‐streak method was easier to interpret and more reproducible. To perform the cross‐streak method, a single colony of a spore‐forming strain was used to inoculate a straight line in the middle of an LB agar plate. After inoculation, the plates were incubated at 30°C for 48 h. After confirmation of a well‐defined growth line, up to five guide lines were drawn on the bottom of the plate perpendicular to the growth line (Figure 2). Overnight cultures were prepared from single colonies of each target strain and were cultured as described previously. Five µL of each overnight culture was spotted in the middle of the corresponding guide lines on each side of the center growth line, and then streaked perpendicular to the growth line, moving first towards the line and then away from it. Controls included plates with target strains but no center growth line. When spore‐forming isolates exerted inhibitory activity in the cross‐streak assay, they were Gram stained to ensure expected morphology (Gram‐positive or Gram‐variable, spore‐forming rods), after which freezer stocks were made.

### 2.4 Taxonomic Identification of Producer Strains

**Figure 3:**
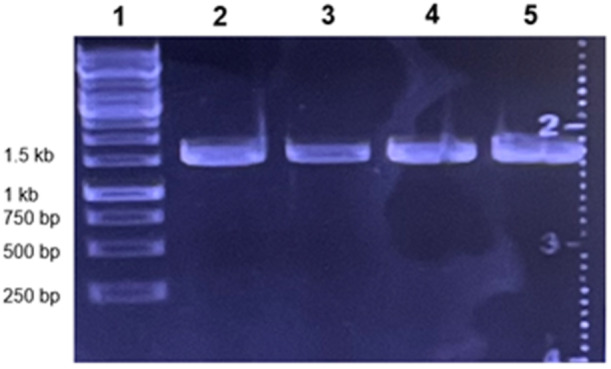
PCR amplification of 16S rRNA gene fragments from representative bacterial isolates. The predicted size of each producer strain amplicon was slightly over 1500 bp, consistent with the ~1550 bp length of the 16S rRNA gene sequence. **1**: ladder*;* **2**: *Aneurinibacillus migulanus* 3.3*;* **3**: *Paenibacillus polymyxa* 9.3 C*;* **4**: *Paenibacillus polymyxa* 9.3 W*;* **5**: *Bacillus mojavensis* 10.4. Similar amplicons were obtained with other selected isolates.

To identify the genus and species of each producer strain, we sequenced the 16S rRNA region and performed a nucleotide BLAST against the NCBI 16S ribosomal rRNA sequence database. Genomic DNA was isolated from single colonies using the Zymo Quick‐DNA Fungal/Bacterial Miniprep Kit (Zymo Research, Catalog No. D6005). The target 16S region was amplified using Q5 Hot Start High‐Fidelity 2X Master Mix (NEB, Catalogue No. M0494S) with the primers GriffF (5′ AGAGTTTGATCCTGGCTCAG 3′) and GriffR (5′ TACGGCTACCTTGTTACGACTT 3′). PCR settings were as follows: 98°C for 30 s; 33 cycles of 98°C for 10 s, 66°C for 20 s, and 72°C for 45 s; 72°C for 2 min; and an infinite hold at 4°C. Negative controls consisted of ddH_2_O in place of genomic DNA, and using the same cycle parameters. The PCR products were run on a gel with a 1 kb ladder to confirm proper length (~1550 bp) (Figure 3) and were cleaned up using the Zymo DNA Clean & Concentrator‐5 kit (Zymo Research, Catalogue No. D4013). Sanger sequencing was performed at Eton Biosciences Inc. (Union, NJ, USA) using the GriffF and GriffR primers, resulting in two sequences of approximately 900 bp. These sequences were aligned to construct the entire 16S rRNA gene, after which NCBI nucleotide BLAST was used to identify the closest genus and species.

### 2.5 Construction of Phylogenetic Tree Using MEGA12

The obtained 16S rRNA sequences were compared with 16S rRNA sequences of *Bacillus*, *Paenibacillus*, and *Aneurinibacillus* available from the GenBank database (www.ncbi.nlm.nih.gov/genbank/). *Pseudomonas aeruginosa* strain ATCC 9999 was included as an outgroup. GenBank accession numbers for these 16S rRNA sequences are listed in Table 2. The phylogenetic tree was constructed in MEGA12 (Kumar et al. 2024) by performing 1000 bootstrap replicates (Felsenstein 1985) using the neighbor‐joining statistical method (Saitou 1987). The evolutionary distances were computed using the Maximum Composite Likelihood method (Tamura et al. 2004) and are in units of the number of base substitutes per site.

**Table 2:** Names and accession numbers of 16S rRNA sequences used in phylogenetic tree.

Strain	GenBank accession number
*Aneurinibacillus migulanus* strain ATCC 9999	NR_115593
*Bacillus velezensis* strain FZB42	NR_075005
*Bacillus cereus* ATCC 14579	NR_074540
*Bacillus halotolerans* strain ATCC 25096	OQ876681
*Bacillus mojavensis* strain IFO 15718	NR_024693
*Bacillus mycoides* strain ATCC 6462	NR_115993
*Bacillus pumilus* strain ATCC 7061	NR_043242
*Bacillus safensis* FO‐36b	NR_041794
*Bacillus subtilis* strain ATCC 6633	AB018486
*Paenibacillus polymyxa* ATCC 842	OR506150
*Paenibacillus alvei* DSM 29	NR_042091
*Paenibacillus amylolyticus* strain NRRL NRS‐290	NR_025882
*Paenibacillus dendritiformis* J47TS5	LC588610
*Paenibacillus profundus* strain SI 79	NR_132304
*Pseudomonas aeruginosa* strain ATCC 15442	AF094718

### 2.6 Illumina Sequencing and Identification of Biosynthetic Gene Clusters Using Antismash

Genomic DNA from *P. polymyxa* 9.3 C, *P. amylolyticus* 9.5, and *P. profundus* M4.5 was isolated from single colonies using the Zymo *Quick*‐DNA HMW MagBead Kit (Zymo Research, Catalog No. D6060). Illumina sequencing was performed at SeqCenter (Pittsburg, PA, USA). Illumina sequencing libraries were prepared using the Illumina DNA Prep kit and IDT 10 bp UDI indices, and sequenced on an Illumina NextSeq. 2000, producing 2 × 151 bp reads. Demultiplexing, quality control and adaptor trimming was performed with bcl‐converter (v3.9.3). Genome sequencing statistics are shown in Table 3.

**Table 3:** Whole‐genome sequencing statistics for producer strain 9.3 C, 9.5, and M4.5.

Isolate	Total read pairs	Total reads (R1 + R2)	Number of contigs	N50	L50	G + C content	Sequence size (bp)	# of putative ORFs
9.3 C	5,256,719	10,513,438	330	351,826	6	45.6%	5,817,989	5543
9.5	4,491,898	8,983,796	329	227,780	10	45.7%	6,419,502	5898
M4.5	5,257,436	10,514,872	513	74,684	27	49.9%	6,653,573	6758

We analyzed the sequenced genomes using antiSMASH (antibiotics and the Secondary Metabolite Analysis Shell) (Blin et al. 2023) in an effort to identify bioactive compound production genes and related operons. Putative ORFs were predicted by RAST (Rapid Annotation using Subsystem Technology) server (Aziz et al. 2008).

### 2.7 Collection and Assessment of Antimicrobial Compounds From Producer Strain Broth Cultures

Producer strains were grown in liquid culture for the purpose of collecting cell‐free supernatant (CFS) containing secreted antimicrobial compounds. A single colony from each producer strain was used to inoculate 5 mL of LB broth in a sterile 15 mL conical tube and grown at 37°C for 24 h while shaking at 175 rpm. One mL from the overnight cultures were transferred into 250‐mL Erlenmeyer flasks containing 99 mL of LB broth and grown under the same conditions for 1–4 days. The cultures were centrifuged at 3837 RCF for 10 min and the supernatants were passed through Nalgene Rapid‐Flow Sterile Disposable Filter Units with 0.2 µm pore size SFCA membranes (Thermo Scientific, Catalog No. 155‐0020). The collected filtrate was filtered again using 10 mL syringes (VWR, Catalog No. 76837‐030) and 0.22 µm pore size CA filters (VWR, Catalog No. 76479‐044) to ensure sterility. Filtrates were kept at 4°C.

The inhibitory activity of producer strain CFS was assessed using a 96‐well microplate growth curve assay. Experimental wells contained 100 µL of CFS or extract, 98 µL of fresh medium, and 2 µL of a target strain culture that had been diluted to OD_600_  =  0.05 immediately before inoculation. Spent supernatant control wells contained 100 µL of *Bacillus anthracis* Sterne strain‐derived CFS, 98 µL of fresh medium, and 2 µL of diluted target strain culture. Positive control wells contained 194 µL of fresh medium, 4 µL of doripenem (0.1 mg/mL) or colistin (0.1 mg/mL), and 2 µL of diluted target strain culture. Negative control wells contained 198 µL of fresh medium and 2 µL of diluted target strain culture. Blank wells contained 200 µL of fresh medium. Prepared microplates were sealed using Breathe‐Easy® membranes (Millipore Sigma, Catalog No. Z380059). The microplate reader was held at 37°C while OD_600_ readings were measured every hour for 24 h with agitation between readings.

### 2.8 Statistical Analysis

Statistical analysis was performed using GraphPad Prism (version 10.6.1). To assess the effects of treatment and time on bacterial growth, two‐way analysis of variance (ANOVA) was conducted with treatment condition and incubation time as fixed factors. Post hoc comparisons were performed using Dunnett's multiple comparisons test to compare each treatment group to the untreated control at each time point. Results were considered statistically significant at *p* < 0.05. Graphs display mean ± standard deviation (SD) from three technical replicates.

## 3 Results

### 3.1 Isolation of Spore‐Forming Bacteria From the Soil

**Figure 4:**
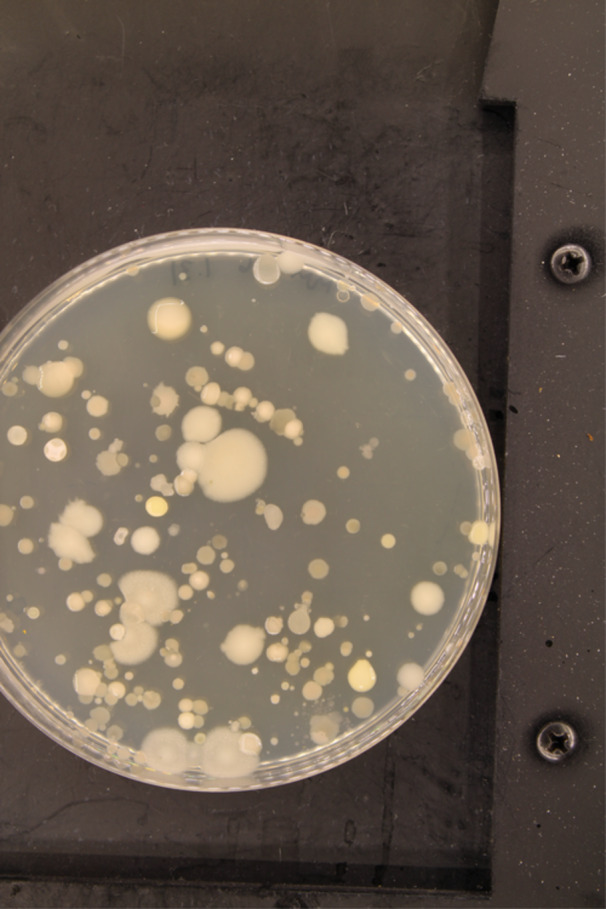
Diluted soil sample spread plate. Soil samples were diluted and spread on LB agar plates. Colonies with unique morphology were picked and tested for antimicrobial activity.

Spore‐forming soil bacteria, such as *Bacillus* and *Paenibacillus*, are known to be prolific producers of antimicrobial compounds (Grady et al. 2016; Olishevska et al. 2019; Celandroni et al. 2016; Caulier et al. 2019; Tran et al. 2022; Sumi et al. 2015). Accordingly, spore‐forming bacteria were selectively isolated from soil samples by heating them to 80°C, thereby eliminating non‐sporulating species. Colonies were chosen for antimicrobial screening primarily based on unique morphologies (Figure 4). An average of five colonies were picked and streaked for isolation from each spread plate, resulting in about 500 isolates used in the antimicrobial screens.

### 3.2 Antibacterial Screens of Soil Isolates Against Clinically Relevant Pathogens

Antibacterial screens were carried out to determine the inhibitory activity of the isolated bacteria. Each of the approximately 500 bacterial isolates were screened using an adapted spread patch method against four target strains: *Staphylococcus aureus, Escherichia coli, Mycobacterium phlei*, and *Pseudomonas aeruginosa*. The first three target strains were chosen because they represent the three main types of bacterial cell envelopes (Gram‐positive, Gram‐negative, and acid‐fast, respectively), while *P. aeruginosa* was chosen because of its intrinsic resistance to many antibiotics (Breidenstein et al. 2011). Of the approximately 500 isolates screened, data were recorded on only 81 of them due to many of the isolates showing either inhibitory patterns similar to isolates already screened, or no inhibition at all. The 81 isolates exhibited a wide range in the strength and spectra of their antimicrobial activity. *S. aureus* was the most affected target strain, being inhibited by 93% (75/81) of the isolates, followed by inhibition of *M. phlei* by 78% (63/81), *E. coli* by 19% (15/81), and *P. aeruginosa* by 12% (10/81).

From this pool of 81 soil isolates, 29 were chosen for further screening. This group consisted of 15 isolates that inhibited either the *E. coli* or *P. aeruginosa* target strains, and 14 isolates that displayed potent antimicrobial activity against the *S. aureus* and/or *M. phlei* target strains but showed no activity against the Gram‐negative target strains (Table 4). The 29 selected producer strains were screened against 10 methicillin‐resistant *Staphylococcus aureus* (MRSA) target strains. Inhibition of the MRSA target strains was common among the 29 producer strains, with 93% (27/29) of the producer strains displaying activity. Inhibitory patterns against MRSA and *S. aureus* were comparable for most of the 27 producer strains, but 81% (22/27) of the producer strains displayed equal to or lower levels of inhibition (on average) against MRSA as compared to *S. aureus*.

**Table 4:** Average zone of inhibition (in mm) of MRSA and other target strains affected by producer strains.

Producer strains	*Staphylococcus aureus* ATCC 6538	*Escherichia coli* ATCC 11229	*Mycobacterium phlei* Presque Isle	*Pseudomonas aeruginosa* ATCC 15442	Methicillin‐resistant *Staph aureus* (MRSA) (average, *n* = 10)
*Bacillus mojavensis* II	6.5	—	—	—	10
*Bacillus cereus* 22	6.5	—	8.5	—	9
*Paenibacillus polymyxa* 4.2	17	10.5	12	6.5	15
*Bacillus subtilis* 16.2	5.5	—	—	—	5.5
*Aneurinibacillus migulanus* 3.3	12	—	13.5	—	12
*Paenibacillus polymyxa* 9.3 C	15.5	9.5	10.5	6	15
*Paenibacillus polymyxa* 9.3 W	17.5	9.5	10.5	4.5	14.5
*Bacillus mojavensis* 10.4	9.5	—	—	—	10
*Paenibacillus profundus* 7.5	11.5	10.5	17.5	8	17
*Paenibacillus amylolyticus* 9.5	—	7.5	—	5	—
*Paenibacillus amylolyticus* 5.6	—	6.5	—	4	—
*Paenibacillus dendritiformis* 6.7	9	8	15.5	6	11
*Paenibacillus dendritiformis* 18.7	11	10.5	16	8.5	6.5
*Bacillus velezensis* 3.8	17.5	—	11	—	13
*Bacillus halotolerans* 5.9	15.5	—	9	—	13
*Bacillus halotolerans* 8.9	15	—	8	—	13
*Paenibacillus alvei* M13	10.5	6.5	—	—	3.5
*Bacillus mycoides* M3.14	15	—	—	—	16
*Bacillus subtilis* H18.1	14.5	—	5	—	14
*Paenibacillus profundus* M4.5	16	9	15	7	18
*Paenibacillus dendritiformis* M4.11	13.5	10	16	8.5	13
*Bacillus subtilis* Y4B19	15	—	6	—	13
*Bacillus subtilis* Y20	7	—	5	—	3.5
*Bacillus pumilus* B3	19	3.5	6	—	19
*Bacillus safensis* 17	13	—	—	—	12
*Bacillus mojavensis* M4.2	11	—	6.5	—	8
*Bacillus pumilus* Y3B7	23	5	6.5	—	21.5
*Bacillus pumilus* 10.9	21.5	5	4.5	—	19
*Bacillus pumilus* M2.12	23	6	5	—	19

Ten of the producer strains (all of which displayed antimicrobial activity against *E. coli* and *P. aeruginosa* in previous screens) were additionally screened against 68 carbapenem‐resistant Enterobacterales (CRE) from the CDC and FDA Antibiotic Resistance (AR) Isolate Banks. Data representing the antimicrobial activity against the CRE isolates are shown in Table 5. CRE isolates #1‐31 were from the CDC Antibiotic Resistance Enterobacterales Carbapenem Breakpoint Panel, while the other 37 CRE isolates came from a variety of other CDC panels and were all categorized as colistin resistant. Of the 10 producer strains that were screened against the CRE isolates, *Paenibacillus profundus* strains 7.5 and M4.5 displayed the most potent and widespread inhibitory activity, showing activity against 95% (65/68) and 97% (66/68) of the CRE isolates, respectively, including representative species from every genus tested. CRE isolates from the *Proteus, Morganella*, and *Providencia* genera showed resistance against every strain except for *P. profundus* strains 7.5 and M4.5. Isolates from the *Escherichia, Klebsiella, Citrobacter, Shigella, Salmonella, Acinetobacter, Enterobacter*, and *Raoultella* genera were frequently inhibited by all 10 producer strains. Substantial inhibition against *Serratia* isolates was less common.

**Table 5:** Average zone of inhibition (in mm) of carbapenem‐resistant Enterobacterales (CRE) target strains effected by producer strains.

Producer strains	*Klebsiella* spp. (*n* = 24)	*Escherichia coli* (*n* = 9)	*Citrobacter* spp. (*n* = 5)	*Shigella sonnei* (*n* = 1)	*Salmonella* Typhimurium (*n* = 1)	*Acinetobacter baumannii* (*n* = 1)
*Paenibacillus polymyxa* 4.2	5.5	10	11	9	7	8.5
*Paenibacillus polymyxa* 9.3 C	6.5	10.5	10	8.5	6	9
*Paenibacillus polymyxa* 9.3 W	6.5	10	9	8.5	5	8.5
*Paenibacillus profundus* 7.5	7	8.5	7	5.5	5.5	12
*Paenibacillus amylolyticus 9.5*	5.5	9.5	9	7	5	8
*Paenibacillus dendritiformis* 6.7	5.5	7.5	6.5	—	—	5.5
*Paenibacillus dendritiformis* 18.7	5	7	4.5	—	—	6
*Paenibacillus alvei* M13	4	5.5	4.5	4.5	—	4.5
*Paenibacillus profundus* M4.5	7.5	8.5	7	6.5	5.5	11
*Paenibacillus dendritiformis* M4.11	8.5	11.5	10	5	4.5	12

	***Enterobacter*** **spp. (*n*** = **4)**	***Serratia marcescens*** **(*n*** = **12)**	***Proteus mirabilis*** **(*n*** = **5)**	***Providencia*** **spp. (*n*** = **2)**	***Morganella morganii*** **(*n*** = **3)**	***Raoultella ornithinolytica*** **(*n*** = **1)**
*Paenibacillus polymyxa* 4.2	5.5	0.5	—	—	—	8
*Paenibacillus polymyxa* 9.3 C	5.5	0.5	—	—	—	8.5
*Paenibacillus polymyxa* 9.3 W	5.5	1	—	—	—	8.5
*Paenibacillus profundus* 7.5	6.5	6.5	4	1	1	9
*Paenibacillus amylolyticus 9.5*	3.5	—	—	—	—	6.5
*Paenibacillus dendritiformis* 6.7	4.5	1	—	—	—	7.5
*Paenibacillus dendritiformis* 18.7	6	0.5	—	—	—	8
*Paenibacillus alvei* M13	1.5	0.5	—	—	—	11
*Paenibacillus profundus* M4.5	7	6.5	4	2.5	2	8.5
*Paenibacillus dendritiformis* M4.11	8.5	1.5	—	—	—	12

### 3.3 16S rRNA Sequencing and Phylogenetic Analysis of Producer Strains

We sequenced the 16S rRNA genes of the 29 producer strains that were chosen for the second round of antimicrobial screening, and used BLASTn to discern their closest identity based on percent identity. Of the 29 producer strains, 58% (17/29) were *Bacillus* species, 38% (11/29) were *Paenibacillus* species, and one was *Aneurinibacillus migulanus* (syn. *Bacillus brevis*) (Table 6).

**Table 6:** Closest species, by 16S rRNA gene similarity, of antimicrobial producing isolates. Identified by BLASTn of 16S rRNA sequence against the NCBI 16S rRNA database.

Producer strain	Closest genus and species	Identity (%)
II	*Bacillus mojavensis* strain D50	99.58
22	*Bacillus cereus* strain SBD1‐8	98.25
4.2	*Paenibacillus polymyxa* strain R 4.5	96.23
16.2	*Bacillus subtilis* strain BJP‐01	98.06
3.3	*Aneurinibacillus migulanus* strain B 7	98.87
9.3C	*Paenibacillus polymyxa* strain C2	98.77
9.3W	*Paenibacillus polymyxa* strain J	98.84
10.4	*Bacillus mojavensis* isolate 5	100
7.5	*Paenibacillus profundus* strain ALP‐11	99.93
9.5	*Paenibacillus amylolyticus* strain KUDC1786	99.59
5.6	*Paenibacillus amylolyticus* strain SQR‐21	98.45
6.7	*Paenibacillus dendritiformis* strain 2022CK‐00834	99.72
18.7	*Paenibacillus dendritiformis* strain 2022CK‐00834	99.66
3.8	*Bacillus velezensis* strain KLP25	99.86
5.9	*Bacillus halotolerans* strain XE48	100
8.9	*Bacillus halotolerans* strain PL‐3 16S	99.93
M13	*Paenibacillus alvei* isolate Paenibacillus B‐LR1	95.04
M3.14	*Bacillus mycoides* strain ZTB1	99.52
H18.1	*Bacillus subtilis* subsp. subtilis strain NM148	99.52
M4.5	*Paenibacillus profundus* strain ALP‐12	99.72
M4.11	*Paenibacillus dendritiformis* strain APT36	99.93
Y4B19	*Bacillus subtilis* strain RTS	99.06
Y20	*Bacillus subtilis* strain IP18	99.82
B3	*Bacillus pumilus* strain DS‐01	99.72
17	*Bacillus safensis* strain NS3	99.71
M4.2	*Bacillus mojavensis* isolate 5	99.86
Y3B7	*Bacillus pumilus* strain 3‐19	99.16
10.9	*Bacillus pumilus* strain DT83	99.51
M2.12	*Bacillus pumilus* strain HN‐10	99.52

**Figure 5:**
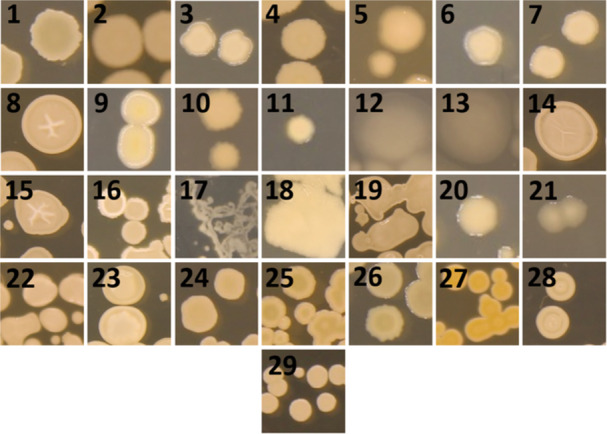
Colony morphologies of selected antimicrobial‐producing strains isolated from the soil around BYU campus. Isolates were grown at 30°C for 48‐72 h. **1**: *Bacillus mojavensis* II*;* **2**: *Bacillus cereus* 22*;* **3**: *Paenibacillus polymyxa* 4.2*;* **4**: *Bacillus subtilis* 16.2*;* **5**: *Aneurinibacillus migulanus* 3.3*;* **6**: *Paenibacillus polymyxa* 9.3 C*;* **7**: *Paenibacillus polymyxa* 9.3 W*;* **8**: *Bacillus mojavensis* 10.4*;* **9**: *Paenibacillus profundus* 7.5*;* **10**: *Paenibacillus amylolyticus* 9.5*;* **11**: *Paenibacillus amylolyticus* 5.6*;* **12**: *Paenibacillus dendritiformis* 6.7*;* **13**: *Paenibacillus dendritiformis* 18.7*;* **14**: *Bacillus velezensis* 3.8*;* **15**: *Bacillus halotolerans* 5.9*;* **16**: *Bacillus halotolerans* 8.9*;* **17**: *Paenibacillus alvei* M13*;* **18**: *Bacillus mycoides* M3.14*;* **19**: *Bacillus subtilis* H18.1*;* **20**: *Paenibacillus profundus* M4.5*;* **21**: *Paenibacillus dendritiformis* M4.11*;* **22**: *Bacillus subtilis* Y4B19*;* **23**: *Bacillus subtilis* Y20*;* **24**: *Bacillus pumilus* B3*;* **25**: *Bacillus safensis* 17*;* **26**: *Bacillus mojavensis* M4.2*;* **27**: *Bacillus pumilus* Y3B7*;* **28**: *Bacillus pumilus* 10.9*;* **29**: *Bacillus pumilus* M2.12.

Morphologies for each are shown in Figure 5. The most identified *Bacillus* species were *B. subtilis* (4 isolates) and *B. pumilus* (4 isolates). Other isolates in the *Bacillus* genera included *B. halotolerans* (2 isolates), *B. mojavensis* (2 isolates), *B. cereus* (2 isolates), *B. mycoides* (1 isolate), *B. velezensis* (1 isolate) and *B. safensis* (1 isolate). Members of the *Paenibacillus* genera included *P. polymyxa* (3 isolates), *P. dendritiformis* (3 isolates), *P. amylolyticus* (2 isolates), *P. profundus* (2 isolates), and *P. alvei* (1 isolate).

**Figure 6:**
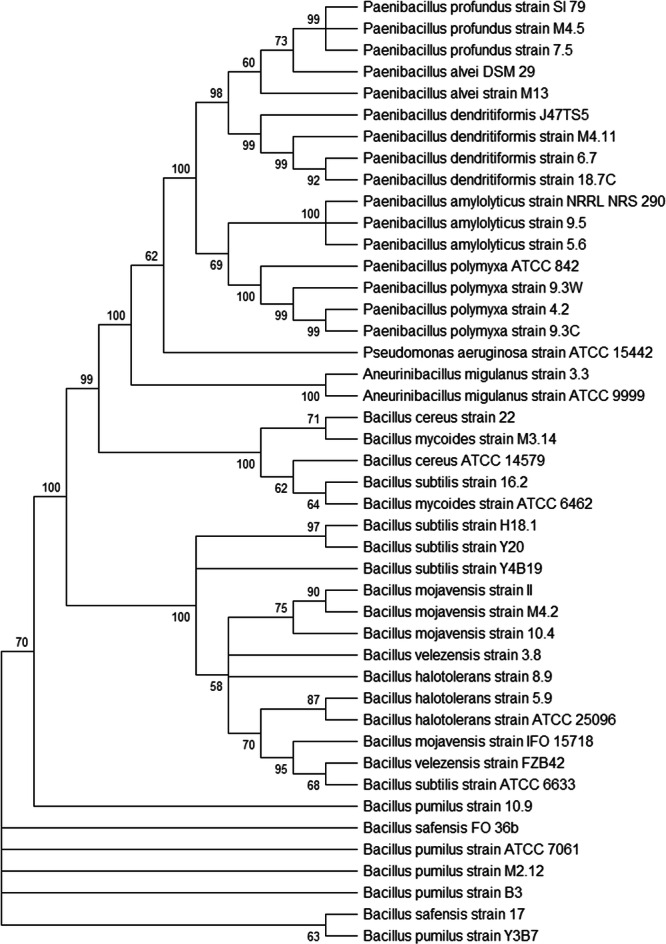
Phylogenetic tree showing the positions of the *Bacillus and Paenibacillus* producer strains relative to other members of those genera. Sequences were aligned using MEGA12, and evolutionary distances were computed using the Maximum Composite Likelihood method.

A phylogenetic tree was constructed using 16S rRNA gene sequences to determine the relationships of the producer strains with *Bacillus*, *Paenibacillus*, and *Aneurinibacillus* species (Figure 6). Many producer strains clustered with the species predicted by BLASTn analysis, particularly among the *Paenibacillus* and *Aneurinibacillus* isolates. In contrast, the *Bacillus* isolates formed looser groupings, suggesting potential inaccuracies in 16S rRNA–based identification that require further analysis.

### 3.4 Biosynthetic Gene Cluster Analysis of Select Producer Strains Using AntiSMASH

Three producer strains were selected for further study, *P. polymyxa* 9.3 C, *P. amylolyticus* 9.5, and *P. profundus* M4.5. These selections were based on their unique inhibitory profiles in the cross‐streak analysis; *P. polymyxa* 9.3 C was able to inhibit the growth of 64/82 target strains, *P. amylolyticus* 9.5 had activity against only Gram‐negative target organisms, and *P. profundus* M4.5 was the most potent producer strain in this study, inhibiting the growth of 80/82 target strains. The potential of the three strains to synthesize antimicrobials was explored using antiSMASH, a bioinformatics tool that predicts putative biosynthetic gene clusters (Blin et al. 2023).

antiSMASH identified 17 regions in the genome of *P. polymyxa* 9.3 C that were associated with the production of antimicrobial compounds (Table 7). The types of detected BGCs were polyketide synthases (PKS), trans‐acyltransferase PKS (trans‐AT PKS), nonribosomal peptide synthases (NRPS), PKS/NRPS hybrids, and a variety of ribosomally synthesized and post‐translationally modified peptides (RiPPs) including a lasso peptide, a proteusin, a ranthipeptide, and two class I lanthipeptides. Of the seventeen regions identified, four showed high similarity to known clusters associated with antimicrobial compounds: region 12.1 and the RiPP paenilan, region 19.1 and the polyketide/nonribosomal peptide (PK/NRP) hybrid fusaricidin, region 26.1 and the nonribosomal peptide (NRP) polymyxin B, and region 29.1 and the NRP tridecaptin. Region 17.1 displayed medium similarly to a known cluster associated with the PK/NRP hybrid paenilipoheptin. There were six regions associated with the biosynthesis of compounds with low similarity to known clusters, namely the PKS cluster aurantinin (region 1.2), the RiPP cluster paeninodin (region 7.2), and the four NRPS clusters marthiapeptide A (region 7.1), tridecaptin (region 14.1), and octapeptin C4 (regions 18.1 and 27.1). The remaining putative biosynthetic clusters (regions 2.1, 7.3, 8.1, 9.1, 11.1, and 15.1) were predicted to drive the biosynthesis of previously unidentified compounds with no known similarity to characterized compounds.

**Table 7:** Results from the antiSMASH analysis of the *P. polymyxa* 9.3 C genome.

Region	Region type	From	To	Size (kb)	Most similar known cluster	Similarity confidence
1.2	TransAT‐PKS, NRPS, T3PKS, PKS‐like	428,457	530,188	101.7	Aurantinin B/C/D (PKS)	Low
2.1	NRPS‐like	269,661	313,356	43.7		
7.1	NRPS	1	32,864	32.9	Marthiapeptide A (Type I NRPS)	Low
7.2	Lasso peptide	89,016	113,120	24.1	Paeninodin (RiPP)	Low
7.3	Proteusin	221,258	241,494	20.2		
8.1	Class I Lanthipeptide	1	16,646	16.6		
9.1	NRPS	1	35,710	35.7		
11.1	Ranthipeptide	30,459	51,880	21.4		
12.1	Class I Lanthipeptide	50,789	77,796	27.0	Paenilan (RiPP)	High
14.1	NRPS	112,605	146,367	33.8	Tridecaptin (Type I NRPS)	Low
15.1	NRPS	105,837	129,757	23.9		
17.1	NRPS, transAT‐PKS, NRPS‐like	1	89,874	89.9	Paenilipoheptin (Type I NRPS + PKS)	Medium
18.1	NRPS, T1PKS	1	33,458	33.5	Octapeptin C4 (Type I NRPS)	Low
19.1	NRPS	1	45,424	45.4	Fusaricidn B (Type I NRPS + PKS)	High
26.1	NRPS	1	24,697	24.7	Polymyxin B (Type I NRPS)	High
27.1	NRPS	1	24,486	24.5	Octapeptin C4 (Type I NRPS)	Low
29.1	NRPS	1	10,076	10.1	Tridecaptin (Type I NRPS)	High

antiSMASH identified 12 regions in the genome of *P. amylolyticus* 9.5 corresponding to antimicrobial compound production (Table 8). The types of detected clusters included trans‐AT PKS/NRPS hybrid, T3PKS, NRPS, class II and IV lanthipeptides, a proteusin, lasso peptides, and one containing a RiPP recognition element (RRE). One of these clusters (region 29.1) had high similarity with the lipopeptide antibiotic colistin. There were three other regions that displayed low similarity to known compounds, namely the PKS/NRPS hybrid bacillomycin D (region 1.1) and the RiPPs paeninodin (region 3.2) and cochonodin I (region 11.1). Eight gene clusters were predicted to be involved in the synthesis of antimicrobial compounds that bear no resemblance to any identified compounds (regions 3.1, 4.2, 5.1, 5.2, 8.1, 20.1, 23.1, and 28.1).

**Table 8:** Results from the antiSMASH analysis of the *P. amylolyticus* 9.5 genome.

Region	Region type	From	To	Size (kb)	Most similar known cluster	Similarity confidence
1.1	TransAT‐PKS, NRPS	202,525	265,827	63.3	Bacillomycin D (Type I NRPS + PKS)	Low
3.1	NRPS, NRPS‐like	115,460	176,597	61.1		
3.2	Lasso peptide	275,005	298,981	24.0	Paeninodin (RiPP)	Low
4.2	T3PKS	147,396	188,553	41.2		
5.1	NRPS	1	22,495	22.5		
5.2	Protuesin	240,131	260,373	20.2		
8.1	NRPS‐like	50,397	94,101	43.7		
11.1	Lasso peptide, ranthipeptide	112,802	152,483	39.7	Cochonodin I (RiPP)	Low
20.1	Class IV Lanthipeptide	101,685	124,435	22.8		
23.1	Class II Lanthipeptide	2309	25,653	23.3		
28.1	RRE‐containing	16,756	38,231	21.5		
29.1	NRPS	1	75,276	75.3	Colistin A/B (Type I NRPS)	High

Nineteen regions associated with the biosynthesis of antimicrobial agents were identified by antiSMASH in the genome of *P. profundus* M4.5 (Table 9). The types of detected clusters were PKS/NRPS hybrid, transAT‐PKS, PKS, NRPS, and RiPPs. Of the 19 regions of interest, only region 47.1 displayed high similarity to a known cluster, the NRP paenibacterin. Additionally, region 30.1 showed medium similarity to the RiPP paenicidin B. Five other regions had low similarity to known antimicrobial compounds, namely the PKS/NRPS hybrid zwittermicin A (region 1.1), the PKS aurantinin (region 71.1), the NRP pelgipeptin (regions 73.1 and 108.1) and the RiPP actinokineosin (region 110.1). The remaining 12 predicted clusters (regions 13.1, 28.1, 45.1, 50.1, 59.1, 63.1, 64.1, 78.1, 83.1, 97.1, 100.1, and 102.1) displayed no similarity to any known BGCs associated with antimicrobial agents.

**Table 9:** Results from the antiSMASH analysis of the *P. profundus* M4.5 genome.

Region	Region type	From	To	Size (kb)	Most similar known cluster	Similarity confidence
1.1	T1PKS, NRPS	246,947	328,042	81.1	Zwittermicin A (Type I NRPS + PKS)	Low
13.1	NRPS	77,794	106,823	29.0		
28.1	T1PKS, NRPS	12,271	70,542	58.3		
30.1	Class I Lanthipeptide	1	23,660	23.7	Paenicidin B (RiPP)	Medium
45.1	Proteusin	37,665	53,018	15.4		
47.1	NRPS	23,513	50,247	26.7	Paenibacterin (Type I NRPS)	High
50.1	TransAT‐PKS, NRPS	1	47,207	47.2		
59.1	TransAT‐PKS‐like, T1PKS	1	39,521	39.5		
63.1	NRPS	1	26,432	26.4		
64.1	NRPS	1	34,956	35.0		
71.1	PKS‐like, transAT‐PKS	1	27,234	27.2	Aurantinin B/C/D (PKS)	Low
73.1	NRPS, NRPS‐like	1	26,933	27.0	Pelgipeptin A/B/C/D (Type I NRPS)	Low
78.1	RiPP‐like	1	11,834	11.8		
83.1	NRPS	1	22,652	22.7		
97.1	NRPS	1	16,080	16.1		
100.1	NRPS	1	15,319	15.3		
102.1	NRPS	1	13,717	13.7		
108.1	NRPS	1	12,625	12.6	Pelgipeptin A/B/C/D (Type I NRPS)	Low
110.1	Lasso peptide	1	11,238	11.2	Actinokineosin (RiPP)	Low

Although many of the predicted clusters may remain cryptic under our culture conditions, these findings support our agar‐based observations that these strains produce antimicrobial compounds.

### 3.5 Extraction and Bioassay of Antimicrobial Compounds From Producer Strains

Having confirmed the inhibitory activity of the producer strains and identified biosynthetic gene clusters in three of them, the next step was to isolate the secreted antimicrobial compounds. To achieve this, the producer strains were cultivated in broth, and the CFS was extracted, which contained any antimicrobial compounds secreted during growth. Ten producer strains that represented a wide range of antimicrobial activities were selected for extraction: *P. polymyxa* 4.2, *P. polymyxa* 9.3 C, *P. profundus* 7.5, *P. amylolyticus* 9.5, *P. dendritiformis* 18.7 C, *B. halotolerans* 5.9, *B. subtilis* H18.1, *P. profundus* M4.5, *P. dendritiformis* M4.11, and *B. pumilus* Y3B7. Nine of these readily secreted antimicrobial compounds in broth cultures, but *P. amylolyticus* 9.5 did not produce detectable levels of antimicrobial compounds under any tested conditions and was therefore excluded from further analysis. Following the same protocol used to obtain the CFS from the eight producer strains, we also extracted the CFS from a liquid culture of *Bacillus anthracis* Sterne strain. This species has not been shown to produce any kind of antimicrobial compound, so the CFS from this strain served as a negative control in this experiment, demonstrating that observed inhibitory activity was not due to a lack of nutrients in spent supernatant or other similar phenomena.

**Figure 7:**
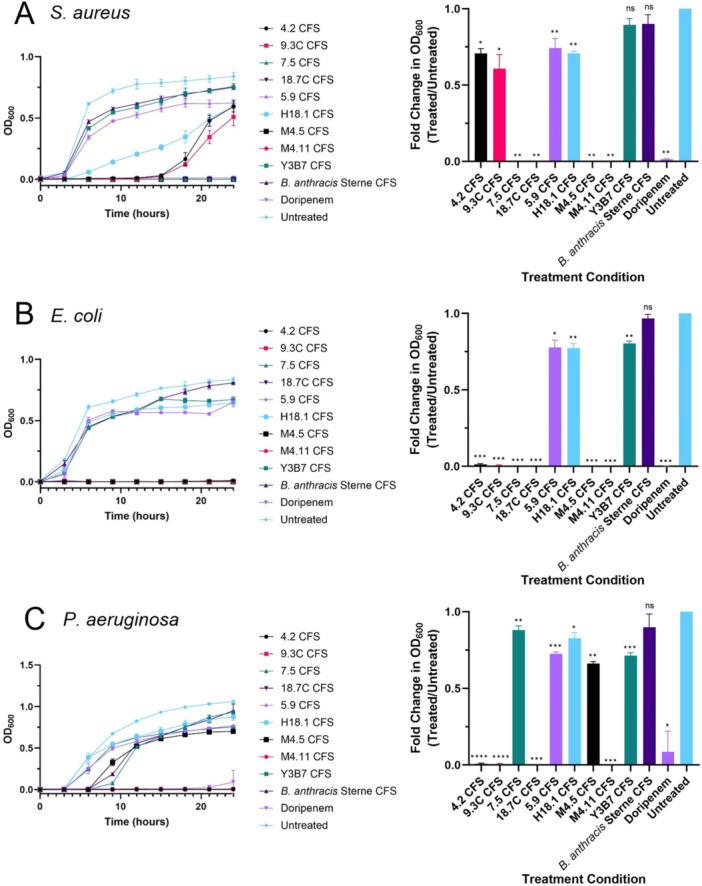
Inhibitory activity of producer strain cell‐free supernatants against target strains. Growth curves (left) and fold changes in growth (right) of: (A) *Staphylococcus aureus*. (B) *Escherichia coli*. (C) *Pseudomonas aeruginosa*. Growth curves depict 24 h of incubation with producer strain supernatants, while fold changes represent hour 24 of that growth period. Data shown as mean ± SD from three technical replicates. Statistical analysis was performed using two‐way ANOVA with Dunnett's multiple comparisons test to compare each treatment group to the untreated control at each time point. ns, not significant; *, **, ***, **** denote *p* < 0.05, *p* < 0.01, *p* < 0.001, and *p* < 0.0001, respectively.

The inhibitory activity of the producer stain CFS was first tested against three of the original target strains, *S. aureus*, *E. coli*, and *P. aeruginosa* (Figure 7). After about 24 h, eight of the nine supernatants caused statistically significant inhibition of the growth of *S. aureus* (Figure 7A). Y3B7 CFS and *B. anthracis* Sterne CFS performed comparably with the untreated control group. Interestingly, although 4.2 CFS and 9.3 C CFS completely inhibited target growth during the first 14 h, their activity declined thereafter, suggesting that the active compound may have degraded over time or that its initial concentration was insufficient to sustain complete inhibition. Following treatment with H18.1 CFS, *S. aureus* exhibited statistically significant growth inhibition at all time points after hour 3, with the usual sigmoidal curve replaced by a nearly flat growth profile. Against *E. coli*, the *Paenibacillus* producer strain supernatants all achieved complete inhibition, while the *Bacillus* producer strain growth curves closely resembled the untreated control (Figure 7B). The growth curves corresponding to the 5.9 CFS, H18.1 CFS, and Y3B7 CFS treatments paralleled the untreated control up to around hour 12. Beyond this point, the untreated control and *B. anthracis* Sterne CFS curves continued to rise, whereas the remaining treatment groups exhibited a plateau in growth. The CFS from the *P. polymyxa* and *P. dendritiformis* producer strains completely inhibited the growth of *P. aeruginosa* (Figure 7C). Although all producer strain supernatants exhibited statistically significant inhibition, five of them had minimal impact on the growth of *Pseudomonas*. Growth was initially slowed following treatment with 7.5 CFS and M4.5 CFS; however, by hour 24, only M4.5 CFS maintained slight inhibition, clustering with Y3B7 CFS and 5.9 CFS just below the untreated control. In contrast, 7.5 CFS and H18.1 CFS showed little to no inhibitory effect by the final time point.

**Figure 8:**
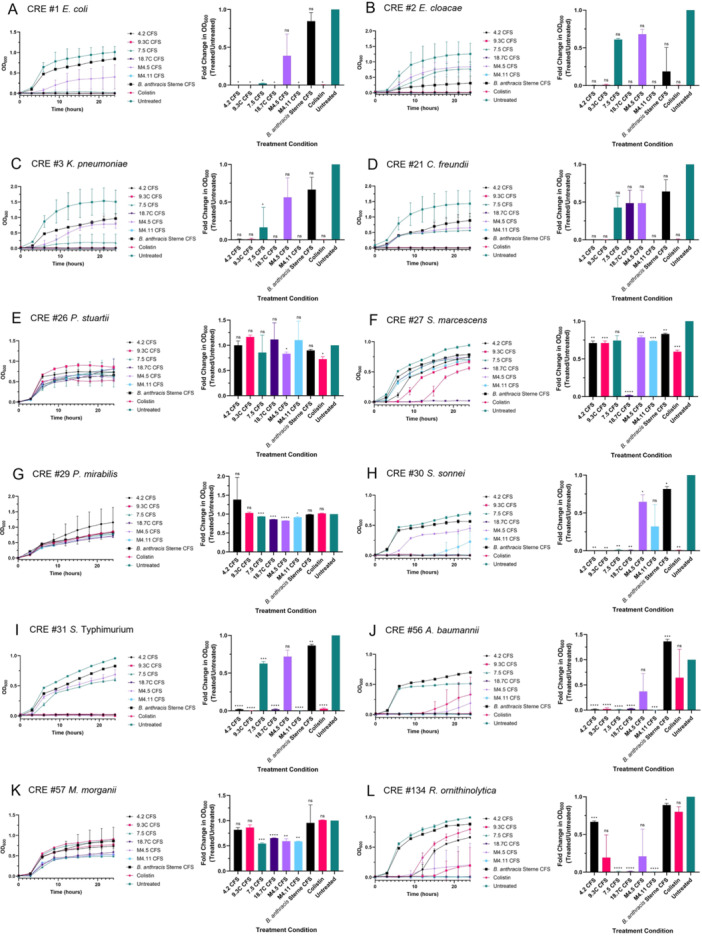
Inhibitory activity of producer strain cell‐free supernatant against CRE target strains. Growth curves (left) and fold changes in growth (right) of: (A) CRE #1 *Escherichia coli*. (B) CRE #2 *Enterobacter cloacae*. (C) CRE #3 *Klebsiella pneumoniae*. (D) CRE #21 *Citrobacter freundii*. (E) CRE #26 *Providencia stuartii*. (F) CRE #27 *Serratia marcescens*. (G) CRE #29 *Proteus mirabilis*. (H) CRE #30 *Shigella sonnei*. (I) CRE #31 *Salmonella* Typhimurium. (J) CRE #56 *Acinetobacter baumannii*. (K) CRE #57 *Morganella morganii*. (L) CRE #134 *Raoultella ornithinolytica*. Growth curves depict 24 h of incubation with producer strain supernatants, while fold changes represent hour 24 of that growth period. Data shown as mean ± SD from three technical replicates. Statistical analysis was performed using two‐way ANOVA with Dunnett's multiple comparisons test to compare each treatment group to the untreated control at each time point. ns, not significant; *, **, ***, **** denote *p* < 0.05, *p* < 0.01, *p* < 0.001, and *p* < 0.0001, respectively.

The six producer supernatants with activity against *E. coli* and/or *P. aeruginosa* underwent additional testing against a select panel of CREs (Figure 8). These target strains included one from each genus listed in Table 5, namely CRE #1 *Escherichia coli*, CRE #2 *Enterobacter cloacae*, CRE #3 *Klebsiella pneumoniae*, CRE #21 *Citrobacter freundii*, CRE #26 *Providencia stuartii*, CRE #27 *Serratia marcescens*, CRE #29 *Proteus mirabilis*, CRE #30 *Shigella sonnei*, CRE #31 *Salmonella* Typhimurium, CRE #56 *Acinetobacter baumannii*, CRE #57 *Morganella morganii*, and CRE #134 *Raoultella ornithinolytica*. Although several panels of the following figure show clear inhibition of target strain growth by the CFS treatment groups at the 24‐h timepoint, some measurements did not reach statistical significance, likely due to high variability within the untreated control group.

CRE #1 *E. coli* was one of the most inhibited CRE target strains that we tested (Figure 8A), with statistically significant inhibition occurring with every treatment except M4.5 CFS and *B. anthracis* Sterne CFS. While treatment with 4.2 CFS, 9.3 C CFS, 18.7 C CFS, M4.11 CFS, and colistin caused observable inhibition of CRE #2 *E. cloacae*, none of those treatments reached statistical significance (Figure 8B). Interestingly, *B. anthracis* Sterne CFS seemed to have notably slowed the growth of CRE #2, and this level of inhibition was not observed with any other target strain. The growth curves of CRE #3 *K. pneumoniae* were like those of CRE #2, except for a greater standard deviation observed with the 7.5 CFS treatment and reduced inhibition by the *B. anthracis* Sterne CFS (Figure 8C).

As noted previously, while none of the treatment groups achieved statistically significant inhibition against CRE #21 *C. freundii*, treatment with 4.2 CFS, 9.3 C CFS, M4.11 CFS, and colistin completely halted bacterial growth (Figure 8D). CRE #26 *P. stuartii* was one of three CRE target strains that showed no observable growth inhibition in response to any of the CFS treatments, as the growth curves for all treatment groups closely resembled that of the untreated control (Figure 8E). However, despite the lack of visible inhibition, statistical analysis revealed that a few treatment groups, including M4.5 CFS, M4.11 CFS, and colistin, achieved modest but statistically significant reductions in growth at the 24‐h time point compared to the control. Apart from 18.7 C, most treatment groups had little to no effect on the growth of CRE #27 *S. marcescens* after 24 h (Figure 8F). Curiously, treatment with 4.2 CFS, 9.3 C CFS, and colistin extended the lag phase of the target strain.

CRE #29 *P. mirabilis* was another CRE that showed no observable growth inhibition by any of the treatments (Figure 8G). Nevertheless, treatment with 7.5 CFS, 18.7 C CFS, M4.5 CFS, and M4.11 CFS still reached statistical significance. Several treatment groups significantly inhibited the growth of CRE #30 *S. sonnei*, including 4.2 CFS, 9.3 C CFS, 7.5 CFS, 18.7 C CFS, and colistin (Figure 8H). M4.11 CFS entirely inhibited *S. sonnei* through hour 15 but slowly lost its inhibitory effect over the next several hours. The results depicted in Figure 8I are comparable to those seen in Figure 8B with *E. cloacae*, except that the growth of CRE #31 *S*. Typhimurium was largely unaffected by *B. anthracis* Sterne CFS.

Through hour 12, growth of CRE #56 *A. baumannii* was significantly arrested by all treatment groups besides *B. anthracis* Sterne CFS (Figure 8J). Starting at hour 12, though, both colistin and M4.5 CFS started to lose their antimicrobial effects. The CRE #57 *M. morganii* growth curves corresponding to treatment with 7.5 CFS, 18.7 C CFS, M4.5 CFS, and M4.11 CFS clustered together slightly below the rest of the curves, all reaching statistical significance at 24 h (Figure 8K). However, growth of this organism was not entirely stopped by any of the treatment conditions. Finally, Figure 8L portrays the growth of CRE #134 *R. ornithinolytica* when treated with the antimicrobial supernatants and controls. Treatment with 4.2 CFS and colistin completely inhibited bacterial growth through hour nine, at which point they followed similar paths and ended up close to the same optical density at hour 24 as the untreated and *B. anthracis* Sterne CFS controls. Similarly, 9.3 C CFS and M4.5 CFS allowed partial growth of *R. ornithinolytica* starting after 9 h. 7.5 CFS, 18.7 C CFS, and M4.11 CFS prevented any growth of this target strain at any time point.

Overall, statistically significant inhibition of many of the target strains was observed when treated with the CFS extracted from the producer strains.

## 4 Discussion

Discovering and developing new antimicrobials to combat drug‐resistant pathogens is a key focus of microbiological research. The rise of antibiotic‐resistant pathogens poses a direct threat to human lives and presents a substantial challenge in managing infectious diseases. We believe that this study indicates the underutilized potential of *Bacillus* and *Paenibacillus* species in the fight against antibiotic resistance.

The results of our study demonstrate that many of the isolated *Bacillus* and *Paenibacillus* strains can inhibit the growth of clinically relevant pathogens, including *Acinetobacter baumannii, Pseudomonas aeruginosa*, MRSA, *Shigella, Escherichia coli*, and *Klebsiella pneumoniae*. Approximately 500 bacterial isolates were screened against four target strains (*S. aureus, E. coli, P. aeruginosa*, and *M. phlei*), and 29 were chosen for further screening and characterization. The genus and species of each of the 29 isolates were determined by sequencing their 16S rRNA gene sequences, and those sequences were mapped to a phylogenetic tree. The antimicrobial activity of each producer strain was measured against a large panel of clinically relevant target bacteria using the cross‐streak method, after which CFSs were extracted from producer strains broth cultures and tested for antimicrobial activity. Finally, whole genome sequencing was performed on three *Paenibacillus* isolates, and their genomes were analyzed for biosynthetic gene clusters using antiSMASH. Together, these findings reveal the ability of these soil isolates to produce antimicrobial compounds (both known and unknown) with the potential that some may prove useful in the fight against antibiotic resistant pathogens.

The initial findings of this study revealed that noticably fewer soil isolates were able to inhibit the growth of Gram‐negative bacteria compared to Gram‐positive bacteria. This observed disparity could be in part explained by unique differences in cell wall architecture such as an outer membrane, LPS, and efflux pumps that are only found in Gram‐negative bacteria. Additionally, many of the antimicrobial peptides (i.e. bacteriocins) produced by *Bacillus*, *Paenibacillus*, and other soil microbes have a relatively narrow spectrum of activity, sometimes only inhibiting closely related bacteria (Abriouel et al. 2011; Jack et al. 1995; Hibbing et al. 2010).

Comparison of the results of the agar‐based and broth‐based antimicrobial screens revealed some minor differences. For example, *P. polymyxa* strains 4.2 and 9.3 C strongly inhibited the growth of *S. aureus* in the cross‐streak assay (see Table 4), but their CFS was only able to inhibit the growth of this bacterium for about 15 h in the microplate assay (see Figure 7A). Similar disparities between activity on an agar plate and CFS activity were seen with *P. profundus* strains 7.5 and M4.5, especially M4.5 which failed to completely inhibit the growth of any of the CRE target strains (see Figures 7 and 8). These discrepancies could be attributed to differential expression of antimicrobial compounds between agar and broth environments. On agar plates, nutrient gradients and direct interspecies interactions often induce stress responses that promote the synthesis of secondary metabolites, whereas the relatively homogeneous and nutrient‐rich conditions of broth cultures may not elicit the same level of compound production (Caro‐Astorga et al. 2020).

In contrast to these observations, *P. dendritiformis* strain 18.7 C displayed substantially stronger inhibitory activity in the broth‐based assay compared to the cross‐streak assay (see Tables 4 and 5; Figures 7 and 8). This discrepancy likely reflects the unique growth behavior of this strain. When inoculated onto agar, *P. dendritiformis* rapidly spreads outward from the inoculation line, eventually covering much of the plate surface. This extensive spreading can obscure or physically overrun the target strain, making it difficult to accurately assess inhibition using the cross‐streak method.

Several *Paenibacillus* strains isolated in this study demonstrated potent broad‐spectrum antimicrobial activity, indicating their potential as sources of new antibacterial agents. In particular, *P. profundus* strains 7.5 and M4.5 showed notable promise. In the cross‐streak assay, both producer strains inhibited the growth of all tested strains of MRSA, as well as *Mycobacterium phlei*, *P. aeruginosa*, and every carbapenem‐resistant Enterobacterales except for CRE #26 *Providencia stuartii* and #519 *Morganella morganii*. The inhibitory effects exerted by 7.5 CFS and M4.5 CFS were not quite as prominent as in the cross‐streak assay but were nonetheless present. The inhibited organisms represent many of those found on the WHO Priority Pathogens list (WHO 2024) as well as five of the six members of the “ESKAPE” pathogens group (*E. faecium, S. aureus, K. pneumoniae, A. baumannii, P. aeruginosa*, and *Enterobacter* spp.). There is a relatively small amount of information on *P. profundus* in the current literature. Aside from its basic characterization as a Gram‐positive, spore‐forming rod‐shaped bacterium, three papers describe the antimicrobial capabilities of *P. profundus* (Romanenko et al. 2013; Kalinovskaya et al. 2013; Tsadila et al. 2021) and two have nothing to do with antimicrobial properties and instead explore the exoelectrogenic properties of this species (Hubenova et al. 2023, 2022). The studies that explored this species' antimicrobial activity described isolating these strains from deep sea sediment (Romanenko et al. 2013; Kalinovskaya et al. 2013) and Greek honey (Tsadila et al. 2021), whereas the strains in this study were isolated from soil around the roots of plants. While *P. profundus* 7.5 and M4.5 had very similar activity profiles (see Tables 4 and 5), their morphologies were distinct (see Figure 5, panels 9 and 20) and their 16S regions shared 98.6% similarity, suggesting that they were distinct strains. However, the perceived difference in morphologies may be explained by the description provided by Romanenko et al. of their novel *P. profundus* strain, about which they said their strain was able to form two stable morphotypes, one that was white and wrinkled and one that was dark yellow and smooth (Romanenko et al. 2013). Thus, while this paper's findings suggest that the *P. profundus* strains are distinct, further genetic and biochemical analyses are needed to confirm this.

The bacterial target strains against which *P. profundus* has been screened for antibacterial activity in previous work are limited. In the most recent study, *S. aureus, P. aeruginosa, S*. Typhimurium, *A. baumannii*, and *C. freundii* were used as target strains (Tsadila et al. 2021). In the other pair of studies, the target strains included *E. coli, E. faecium, S. aureus, S. epidermidis, B. subtilis*, and *Xanthomonas* sp. pv*. badrii* (Romanenko et al. 2013; Kalinovskaya et al. 2013). In this study, *P. profundus* 7.5 and M4.5 were tested against 15 genera of bacteria, encompassing all three major cell envelope types and exhibiting various levels of antibiotic resistance. Additionally, the results of the antibacterial screens performed by Tsadila et al (Tsadila et al. 2021) differ slightly from the results in the current study. Antimicrobial activity of their *P. profundus* strain was observed only against *S*. Typhimurium and *C. freundii*, while our strains had activity against all five of their target strains. Thus, we believe that this study contains the most comprehensive panel to date for antimicrobial activity screening of *P. profundus* strains.

The results of the antibacterial screens performed previously have both similarities and differences to those of our study. In the screens performed by Tsadila et al. the antimicrobial activity of their *P. profundus* strain was observed against *S*. Typhimurium and *C. freundii* but not *S. aureus, P. aeruginosa*, or *A. baumannii* (Tsadila et al. 2021), while our strains had activity against all five of these target strains. Kalinovskaya et al. described the isolation of a novel heptapeptide and a known isocoumarin antibiotic from a *P. profundus* strain, and observed that the heptapeptide inhibited only tested Gram‐positive target strains, while the isocoumarin was active against both Gram‐positive and Gram‐negative isolates (Kalinovskaya et al. 2013). The observed antimicrobial activity of the isocoumarin antibiotic was similar to what we observed from our *P. profundus* strains. Interestingly, the antiSMASH analysis conducted in this study did not reveal any gene clusters in *P. profundus* M4.5 that are similar to known isocoumarin antibiotic clusters (see Table 9). This absence could reflect several possibilities. First, the genes responsible for isocoumarin biosynthesis in this strain may differ substantially in sequence or organization from currently annotated clusters, preventing antiSMASH from recognizing them. Alternatively, the relevant genes could be fragmented across multiple contigs due to genome assembly limitations or low sequencing coverage, making cluster prediction incomplete. Nonetheless, in *Bacillus* spp., isocoumarins are synthesized via hybrid polyketide synthases‐nonribosomal peptide synthases (PKS‐NRPS) (Li et al. 2015; Iqbal et al. 2023), and two such PKS‐NRPS clusters were identified by antiSMASH in strain M4.5 (regions 1.1 and 28.1, see Table 9). This connection could help explain the similar antimicrobial activity results seen between the studied strains. Taken together, the similarities and differences in the antimicrobial activity observed in our *P. profundus* strains compared to previous studies highlight the potential diversity of bioactive compounds produced by this species, and the identification of PKS‐NRPS clusters in our strains provides a possible explanation for these findings.

Several other *Paenibacillus* strains isolated during this study demonstrated potent antibacterial activity, especially against the carbapenem and colistin resistant Enterobacterales. Three of these isolates were strains of *P. polymyxa* (4.2, 9.3C, and 9.3W). *Paenibacillus polymyxa* has historically been the most thoroughly studied *Paenibacillus* species due to its prolific production of antimicrobial compounds such as polymyxins (Stansly and Schlosser 1947; Rabanal and Cajal 2017) and fusaricidins (Kajimura and Kaneda 1996). Thus, it was not surprising to find that several of the most potent producer strains belonged to this species. antiSMASH predicted that 9.3 C carries three biosynthetic gene cluster with high similarity to clusters associated with known antimicrobials, namely paenilan (region 12.1), polymyxin B (region 26.1), and tridecaptin (region 29.1) (see Table 7). Paenilan is a ribosomally synthesized and post‐translationally modified peptide (RiPP) that is known to have activity against Gram‐positive organisms (Park et al. 2017), and polymyxins and tridecaptins are nonribosomal peptides (NRPs) that primarily target Gram‐negative organisms (Mohapatra et al. 2021; Ledger et al. 2022; Cochrane et al. 2016; Li and Velkov 2019; Lohans et al. 2014). These findings suggest that *P. polymyxa* 9.3 C may inhibit *S. aureus* through the production of paenilan (Table 4; Figure 7A), while its activity against Gram‐negative bacteria is likely mediated by polymyxin B and/or tridecaptin (Tables 4 and 5; Figures 7B and 8). Interestingly, 9.3 C CFS exhibited a stronger inhibitory effect than colistin against both CRE #56 *A. baumannii* and CRE #134 *R. ornithinolytica*. Both of these target strains are designated as colistin‐resistant by the CDC & FDA AR Isolate Bank. Because resistance to colistin typically confers cross‐resistance to polymyxin B (Shahzad et al. 2023; Li et al. 2005; El‐Sayed Ahmed et al. 2020), this observation suggests that an alternative antimicrobial compound like tridecaptin may be responsible for the observed inhibition. Further investigation into the expression and regulation of the biosynthetic gene clusters in *P. polymyxa* 9.3 C will be essential to determine which compounds are actively produced and contributing to these effects.

In addition, three of our strains belonged to the species *P. dendritiformis*, which is also known for its antimicrobial activity (Be'er et al. 2009; Jangra et al. 2018; Sadiqi et al. 2022; Mena et al. 2023). Another particularly interesting strain from this study was *P. amylolyticus* 9.5, which was found to only inhibit Gram‐negative organisms. Genome analysis by antiSMASH predicted that this strain was capable of producing colistin (also known as polymyxin E) (see Table 8), which is supported by the current literature (Decrescenzo Henriksen et al. 2007) and would explain the observation that it only inhibits Gram‐negative organisms. Despite the seven other BGCs predicted to be encoded by this strain, we were unable to find any other research on antimicrobial compound production by *P. amylolyticus* aside from colistin, suggesting a need for further research of this organism. Lastly, we found one strain of *P. alvei* that demonstrated both unique morphological characteristics (see Figure 5, panel 17) as well as limited antimicrobial activity. This activity is well supported by the current literature (Anandaraj et al. 2009; Alkotaini et al. 2013; Pajor 2020a, 2020b).

Taken together, this work demonstrates the ample antibacterial abilities of 29 soil isolates against clinically relevant pathogens. While some of these antimicrobial‐producing bacterial species have been extensively studied, we believe that several of the isolates mentioned in this study (including *P. profundus* and *P. dendritiformis*) have untapped potential as producers of new antimicrobials. Work is currently underway to isolate and characterize potentially novel compounds synthesized by these organisms.

## 5 Conclusions

This study highlights the antimicrobial potential of soil‐derived *Bacillus* and *Paenibacillus* species, demonstrating their ability to inhibit clinically significant pathogens, including carbapenem‐resistant Enterobacterales (CRE) and methicillin‐resistant *Staphylococcus aureus* (MRSA). Notably, *Paenibacillus profundus* strains exhibited potent broad‐spectrum activity, demonstrating their potential as sources of novel antimicrobial agents. Genome mining revealed biosynthetic gene clusters associated with nonribosomal peptide synthetases (NRPSs), polyketide synthases (PKSs), and ribosomally synthesized and post‐translationally modified peptides (RiPPs), further supporting the potential for novel bioactive compound production. While these findings appear promising, they must be further substantiated by additional in vitro validations.

## Author Contributions

**Michael Moran:** conceptualization (equal), investigation (lead), data curation (lead), formal analysis (lead), funding acquisition (lead), methodology (lead), validation (equal), visualization (lead), writing – original draft (lead), writing – review and editing (equal), project administration (lead). **Hogan Turner:** investigation (supporting), data curation (supporting), formal analysis (supporting), validation (equal), visualization (supporting), writing – original draft (supporting). **Joseph Yanchar:** investigation (supporting), data curation (supporting), formal analysis (supporting), validation (equal), writing – original draft (supporting). **Joshua Preece:** investigation (supporting), data curation (supporting), formal analysis (supporting), validation (equal), writing – original draft (supporting). **Gene Ahlborn:** conceptualization (supporting), methodology (supporting). **Richard Robison:** conceptualization (equal), supervision (lead), data curation (supporting), methodology (supporting), visualization (supporting), writing – review and editing (equal), resources (lead).

## Ethics Statement

The authors have nothing to report.

## Conflicts of Interest

The authors declare no conflicts of interest.

## Data Availability

All sequence data is available in GenBank, and all other data generated or analyzed during this study are included in this published article.
